# Shift patterns of internal bacterial communities across five life stages of laboratory-maintained *Eremobelba eharai* (Acari: Oribatida: Eremobelbidae)

**DOI:** 10.3389/fmicb.2025.1512653

**Published:** 2025-03-12

**Authors:** Yu Chen, Jun Chen

**Affiliations:** ^1^Key Laboratory of Zoological Systematics and Evolution, Institute of Zoology, Chinese Academy of Sciences, Beijing, China; ^2^College of Life Sciences, University of Chinese Academy of Sciences, Beijing, China

**Keywords:** microbial communities, oribatid mites, developmental stages, bacterial diversity, functional potential

## Abstract

Microbial communities play a crucial role in the physiological characteristics, adaptability, and reproductive success of arthropods. However, the patterns and functions of microbial variation across different life stages of mites remain poorly understood. In this study, we used high throughput Illumina sequencing of the 16S rRNA gene to investigate the composition and functional potential of bacterial communities in five life stages (larva, protonymph, deutonymph, tritonymph, and adult) of the oribatid mite *Eremobelba eharai*. We found significant differences in bacterial diversity and community structure across the different life stages despite being fed the same diet. The bacterial diversity was highest at the protonymph stage and lowest at the tritonymph stage. Beta diversity analysis indicated distinct bacterial community compositions among the different life stages. Bacteroidota, Proteobacteria, and Firmicutes dominated the bacterial communities throughout the host's life cycle. Key bacterial genera, such as *Bacillus, Streptomyces, Achromobacter*, and *Tsukamurella*, showed significant differences in abundance across the different life stages. Predicted functional profiles revealed substantial changes in metabolic pathways, which may reflect changes in the nutritional needs of *E. eharai* during its developmental process. PICRUSt prediction results also showed that in most KEGG pathways, the larval and adult stages consistently maintain similar relative abundances of bacteria. Different stages such as the deutonymph and adult stages show consistent differences in the “biosynthesis of other secondary metabolites” and “glycan biosynthesis and metabolism” pathways. This study provides new insights into the dynamic changes of bacterial communities within oribatid mites and lays a foundation for further research on the interactions between oribatid mites and microorganisms.

## 1 Introduction

Interactions between microbes and their hosts play a crucial role in the process of animal development and environmental adaptation. This is particularly evident in arthropods, whose digestive or symbiotic systems are colonized by diverse microbial communities (Dillon and Dillon, 2004; Shapira, 2016). Microbial communities play a critical role in the physiological traits, life history characteristics, adaptability to the environment, and reproductive success of arthropods. Moreover, interactions with microbes significantly impact the health, survival, and behavior of the hosts, highlighting the key role of microbial symbionts in their ecology and evolution (Engel and Moran, 2013; Moran et al., 2008; Santos-Garcia et al., 2014). Specifically, microbes may enhance the adaptability of their hosts through various means, including bolstering defenses against parasites and pathogens, modulating mating and reproductive systems, extending lifespan, aiding in the digestion and absorption of nutrients, as well as directly providing nutritional compounds (Arbuthnott et al., 2016; Dillon and Dillon, 2004; Douglas, 2015; Engl and Kaltenpoth, 2018; Grenier and Leulier, 2020; Noman et al., 2021; Shamjana et al., 2024).

Arthropods are the most diverse and abundant branch of the Animal Kingdom, and their internal microbial communities exhibit remarkable diversity and functional complexity. In soil ecosystems, the microbial symbionts of arthropods are particularly important, as they mediate interactions between the host and its surrounding environment, facilitating processes such as detritus decomposition and nutrient cycling (Douglas, 2015; Engel and Moran, 2013). This diversity and complexity highlights the need for in-depth research on a broad range of species, especially soil-dwelling arthropods, which play pivotal roles in maintaining soil health and ecosystem functionality (Anderson, 1988; Haq, 2019). However, despite substantial research demonstrating that soil arthropods harbor rich microbial communities (Agamennone et al., 2015; Ladygina et al., 2009; Thakuria et al., 2010), the functional roles and mechanisms of these microbial associations remain largely unexplored.

Oribatid mites occupy a significant proportion within the composition of soil fauna diversity, representing one of the most widely distributed, diverse, and abundant groups in soil ecosystems. Research has found that in temperate forests, the number of oribatid mite species living in a 1 m^2^ area of soil can reach up to 100–150 species, with individual counts peaking at up to 200,000 (Luxton, 1991; Maraun and Scheu, 2000; Wallwork, 1983). Microorganisms play multiple roles throughout the lifecycle of their arthropod hosts, and the ecology of these symbionts remains poorly understood in many systems (Funkhouser and Bordenstein, 2013; Kohl et al., 2017; Kohl and Carey, 2016; Macke et al., 2017; Oliver et al., 2013). There are very few reports on the response trends of oribatid mite microbiota to environmental changes, with only a limited number of studies focusing on gut microbiota, and current research is restricted to adult mites (Gong et al., 2018; Renker et al., 2005; Sanchez-Chavez et al., 2023; Siepel and Deruiterdijkman, 1993). Moreover, the differences in microbial composition across different life stages of mites are not been well understood. It is very difficult to obtain all immature stages of oribatid mites in the field, and their identification is equally difficult. Most oribatid mites have complex feeding habits and span multiple trophic levels (Maraun et al., 2023), and their exact natural food sources remain unclear. Consequently, artificial rearing is also challenging. As a result, the patterns of microbial changes within oribatid mites at different life stages and their roles have been difficult to assess and remain unclear. *Eremobelba eharai* (Chen and Gao, 2017) (*Oribatida: Eremobelbidae*) is a common oribatid mite that occurs in northern China, with a lifecycle that includes egg, larval (L), protonymph (PN), deutonymph (DN), tritonymph (TN), and adult (A) stages. *E. eharai* reproduces parthenogenetically, has a relatively short lifecycle (approximately 60 days), and can survive on a diet of dry yeast alone in the laboratory. Each female mite can lay 5–6 eggs at a time, making it a suitable subject for laboratory rearing. In this study, we used yeast as a standardized food source to explore *E. eharai* gut microbiota composition under controlled conditions. Furthermore, by referencing other arthropods (Meng et al., 2019; Yao et al., 2019), we hypothesize that the composition and structure of microbial communities in *E. eharai* may also change throughout its lifecycle to meet the nutritional and physiological demands of the host during development.

This study utilized high-throughput Illumina sequencing technology for 16S rRNA genes to measure and compare the internal bacterial community composition and relative abundance across five life stages (including larval, protonymph, deutonymph, tritonymph, and adult) of *E. eharai*, when fed a diet of yeast. The data obtained was used to predict and analyze the functions of significant microbes detected in *E. eharai*. Our study reveals for the first time the pattern of bacterial community changes in different stages of the life cycle of *E. eharai* when fed a diet of yeast.

## 2 Materials and methods

### 2.1 Cultivation of *Eremobelba eharai*

Samples were collected from the litter and soil mixture of a pine tree in Huangcaowan Country Park, Chaoyang District, Beijing City, China (40°0′8.15″N, 116°25′50.29″E, 41.20m) on August 10^th^ 2021. The live mites were extracted using Tullgren funnels and placed into plastic jars (250 mL) with wet cotton at the bottom. Approximatley 300 adult individuals were collected. Some specimens were mounted on slides with Hoyer's medium for identification, and morphological observations. A Leica DM2500 microscope was used to identify the *Eremobelba eharai* (Chen and Gao, 2017) specimens. The remaining live *E. eharai* were reared in several plastic jars (250 mL), with gypsum powder/activated charcoal mixture (9:1) at the bottom (Bruckner et al., 2018), and lids with needle size perforations to allow air exchange. Mites were reared in the dark at 25 ± 3°C and 80% humidity. *E. eharai* were fed once a week with dried yeast and containers were cleaned of uneaten food to avoid growth of mycelium.

### 2.2 *E. eharai* sample preparation

Adult mites obtained from the field were allowed to lay eggs from which ten *E. eharai* larvae of the same generation were chosen for downstream testing. To ensure age synchronization of the larvae, we collected eggs laid within a 24-h period and incubated them under the same temperature and humidity conditions. After hatching, newly emerged larvae were immediately transferred to new rearing containers to ensure that all experimental individuals were at the same developmental stage. Larvae were individually placed in separate rearing containers. After reaching adulthood, all individuals laid eggs that hatched, confirming parthenogenesis. Subsequently, all adult mite samples originated from eggs of the same generation that hatched within a 24-h period. Some of the adult mites were used for molecular experiments, while the remaining individuals were continuously reared until they laid eggs. After hatching, newly emerged larvae were immediately transferred to new rearing containers. As they developed into each successive stage, they were transferred to new containers accordingly. At each developmental stage, a subset of individuals was collected for molecular experiments, while the remaining individuals were reared to the next stage. These samples from five life stages, including larval (L), protonymph (PN), deutonymph (DN), tritonymph (TN), and adult (A). Given that the different life stages of mites differ considerably in size, the number of individuals samples used for the DNA extraction for each stage varied. This was done in order to ensure sufficiently high DNA yields for downstream sequencing (See details in Supplementary Methods). Four replicates were processed for each life stage. To avoid interference from surface microbial DNA, each sample was placed into a 1.5 mL sterile centrifuge tube, suspended in 1 mL of 5% sodium hypochlorite (NaOCl) and treated twice with fresh solution, vortexing for 1 min per step. This step was followed by three washes with 1 mL of sterile water (1 min/step), using three new 1.5 mL sterile centrifuge tube for each step (Juds et al., 2023).

### 2.3 DNA extraction and PCR amplification

The total microbial genomic DNA was extracted using the TIANamp Micro DNA Kit (Tiangen Biotech, Beijing, China) according to the manufacturer's protocol. The quality and concentration of DNA were determined by 1.0% agarose gel electrophoresis and a NanoDrop2000 spectrophotometer (Thermo Scientific, United States). DNA extractions were stored at −80°C until ready to use. The hypervariable region V3-V4 of the bacterial 16S rRNA gene was amplified using primer pairs 341F (5′-CCTACGGGNGGCWGCAG-3′) and 806R (5′-GGACTACHVGGGTWTCTAAT-3′), by GeneAmp^®^ 9700 (BIO-RAD, USA). The PCR reaction mixture contained 10 μL of 2 × Pro Taq, 0.8 μL of each primer (5 μM), 10 ng of template DNA, and ddH_2_O to a final volume of 20 μL. PCR amplification conditions were as follows: 95°C for 3 min (initial denaturation), followed by 29 cycles of 95°C for 30 s (denaturation), 53°C for 30 s (annealing), 72°C for 45 s (extension), this cycling was followed by a final extension at 72°C for 10 min, and ending with a hold at 10°C. The PCR products were extracted from a 2% agarose gel, purified using the PCR Clean-Up Kit (YuHua, Shanghai, China) according to the manufacturer's instructions, and quantified with Qubit 4.0 (Thermo Fisher Scientific, USA). The libraries were constructed using the NEXTFLEX Rapid DNA-Seq Kit (Bioo Scientific, Austin, TX, USA) by ligating adapter sequences to the target DNA regions through PCR amplification. The purified amplicons were pooled in equimolar amounts and paired-end sequenced on an Illumina PE300 platform (Illumina, San Diego, USA) at Majorbio Bio-Pharm Technology Co. Ltd. (Shanghai, China). Sequencing reads were demultiplexed using unique barcodes to accurately assign reads to their respective samples. The raw sequencing reads were deposited into the NCBI Sequence Read Archive (SRA) database with accession no. PRJNA1126081.

### 2.4 Sequence analysis

Paired-end raw sequencing reads were first subjected to quality control using fastp v0.19.6 (Chen et al., 2018). The reads were then merged with FLASH v1.2.11 (Magoc and Salzberg, 2011). Reads with quality scores below 20 at the 3' end and shorter than 50 bp, as well as sequences containing ambiguous bases (N) were discarded. The reads were then merged with FLASH v1.2.11 (Magoc and Salzberg, 2011). Paired reads were combined into single sequences with a minimum overlap of 10 bp and a maximum mismatch rate of 0.2 in the overlapping region. No barcode mismatches were allowed, and primers could have up to 2 mismatches. UPARSE v11 (Edgar, 2013) software was used to cluster operational taxonomic units (OTUs) at a 97% similarity threshold, with chimera removal. To control for sequencing depth variations in alpha and beta diversity analyses, all samples were rarefied to 52,713 sequences. Taxonomic annotation of OTUs was conducted using the RDP classifier v2.2 (Wang et al., 2007) against the Silva 16S rRNA gene database v138 with a confidence threshold of 0.7. Functional predictions for the 16S rRNA gene sequences were made using PICRUSt2 v2.2.0 (Douglas et al., 2020).

### 2.5 Bioinformatics analysis

Alpha diversity indices at the OTU level were calculated using Mothur v1.30.1 (Schloss et al., 2009). This included the Shannon diversity index, Simpson's index, Berger-Parker dominance index, Observed richness (Sobs), Abundance-based Coverage Estimator (ACE), and Chao1 richness estimator. The R package “VennDiagram” (Zaura et al., 2009) was employed to create a Venn diagram displaying shared and unique OTUs, focusing solely on OTU occurrence regardless of relative abundance. Beta diversity analysis was performed to examine structural changes in microbial communities across life stages, utilizing Bray-Curtis dissimilarity with the Vegan v2.5-3 package, and visualized through a heatmap at the class level (Anderson, 2001). Hierarchical clustering at the genus level was conducted using the Unweighted Pair Group Method with Arithmetic Mean (UPGMA), while Principal Coordinates Analysis (PCoA) (Ramette, 2007) was performed at the OTU level. Similarity analysis (ANOSIM) using the “vegan” R package was used to assess the significance of microbial community differences among groups.

## 3 Results

### 3.1 Sequencing data of bacterial communities throughout the life stages of *E. eharai*

Using the Illumina MiSeq platform targeting 16S rDNA gene amplicons, quantitative analysis of bacterial composition was performed on larval, protonymph, deutonymph, tritonymph, and adult stages. Sequencing generated 1,585,514 high-quality reads for the bacterial 16S rRNA gene with an average length of 420 bp (Supplementary Table S1). The number of high-quality valid sequences per sample ranged from 52,713 to 69,729 (Supplementary Table S1). Rank-abundance curves (Supplementary Figure S1A) indicated that most OTUs in bacterial communities were rare species, suggesting a concentrated species distribution based on curve width and smoothness. Rarefaction curves (Supplementary Figure S1B) showed an accumulation trend approaching saturation for OTUs, indicating that the sampling was sufficient to effectively describe and represent the diversity of the bacterial community.

### 3.2 Diversity of bacterial communities throughout the life stages of *E. eharai*

**Figure 1:**
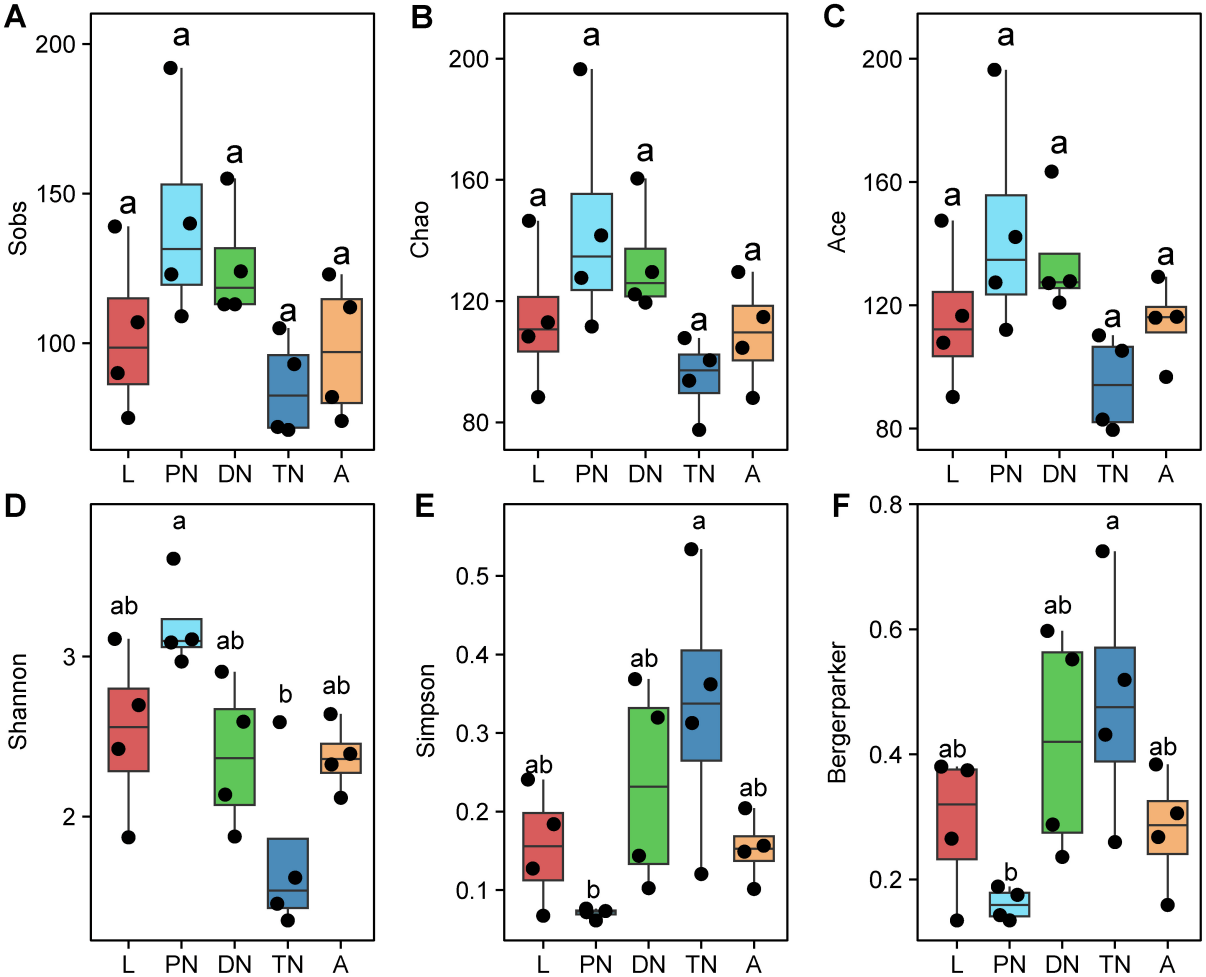
Alpha diversity of bacterial communities across five life stages of E. eharai*. The observed species Sobs **(A)**, Chao **(B)**, and Ace **(C)** indices for species richness, Shannon **(D)**, Simpson **(E)**, and Berger-Parker **(F)** indices for bacterial diversity were applied. All *n* = 4. Different lowercase letters denote significant differences between different life stages (*P* < 0.05, ANOVA with *post-hoc* Tukey HSD Test). Plots showed means ± standard error of mean (SEM). *L, PN, DN, TN, and A indicated the larva, protonymph, deutonymph, tritonymph, and adult stages, respectively.

To identify the diversity of bacteria in larva, protonymph, deutonymph, tritonymph and adult *E. eharai*, four indices were used, including observed species (Sobs), Chao and Ace for species richness, Shannon, Simpson and Berger-Parker for bacterial diversity. The species richness indices (Sobs, Chao and Ace) showed no significant difference among any stage (Figures 1A–C). The Shannon index of showed that the highest diversity in the protonymph stage, while the Simpson and Berger-Parker indices showed the lowest diversity to be in the protonymph stage (*P* < 0.05, ANOVA with *post-hoc* Tukey HSD Test). Subsequently, the Shannon index dropped to its lowest point, while the Simpson and Berger-Parker indices reached their peak in the tritonymph stage (Figures 1D–F).

### 3.3 Differences in composition of the bacterial communities throughout the life stages of *E. eharai*

The Venn diagram analysis and OTU classification revealed that all life stages of *E. eharai* shared 83 bacterial OTUs belonging to five main phyla (Supplementary Table S2). A total of eight main phyla were detected in all samples across all life stages (Supplementary Table S3). The phylum Bacteroidota was one of the predominant phyla identified at different stages, with the highest relative abundance in the adult stage, the lowest in the deutonymph stage. Proteobacteria and Firmicutes are the other two prominent phyla, with the highest relative abundances in the adult stage and tritonymph stage, respectively (Supplementary Figure S2A; Supplementary Table S3). At the class level, the top five taxon in all life stages are Bacteroidia in the phylum Bacteroidota, Bacilli in the phylum Firmicutes, Actinobacteria in the phylum *Actinobacteriota, Alphaproteobacteria* and *Gammaproteobacteria* in the phylum *Proteobacteria* (Supplementary Figure S2B; Supplementary Table S3).

**Figure 2:**
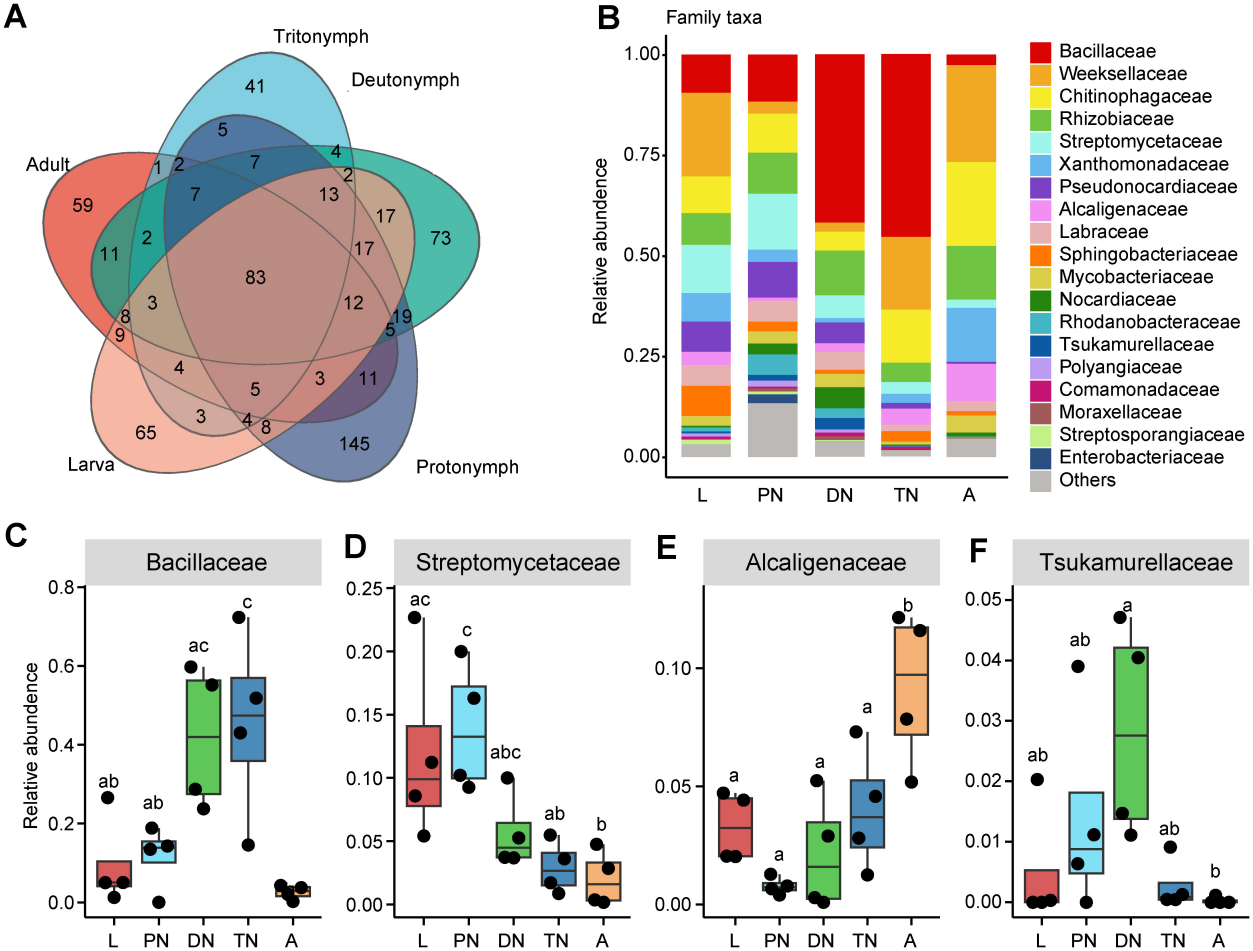
Relative abundances of bacterial families across five life stages of *E. eharai*. **(A)** Overlap of OTUs between different life stages. **(B)** Whole profiles of the relative abundances of the families in each life stage; only taxa ranked in the top 10 in relative abundance in at least one sample were analyzed. **(C–F)** Comparisons of the relative abundances of major families across the larva (L), protonymph (PN), deutonymph (DN), tritonymph (TN), and adult (A) stages; all *n* = 4; different lowercase letters denote significant differences between different life stages (*P* < 0.05, ANOVA with *post-hoc* Tukey HSD Test); plots showed means ± (SEM).

**Figure 3:**
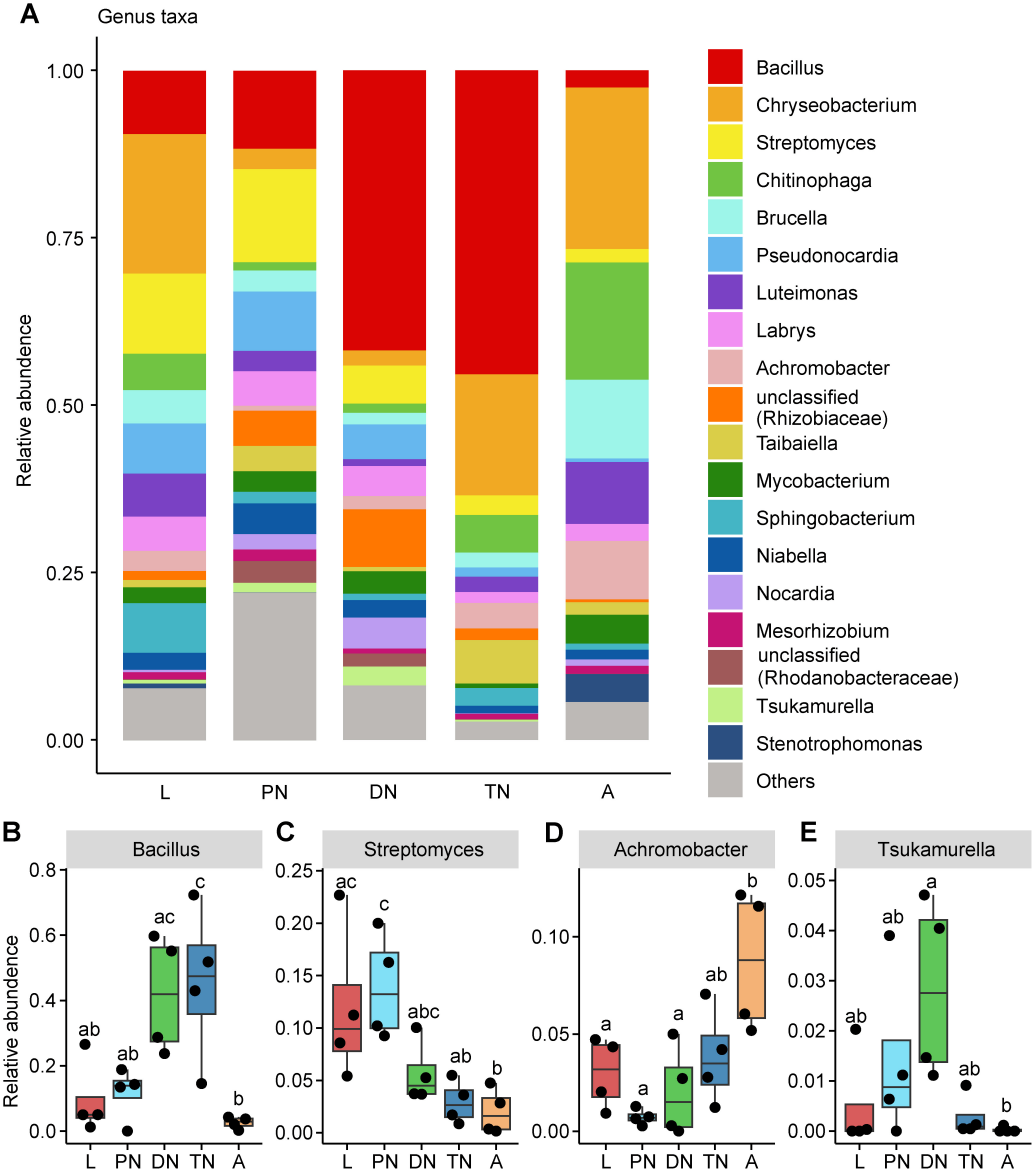
Relative abundances of bacterial genera across five life stages of *E. eharai*. **(A)** Whole profiles of the relative abundances of the genera in each life stage; only taxa ranked in the top 20 in relative abundance in at least one sample were analyzed. **(B–E)** Comparisons of the relative abundances of major genera across the larva (L), protonymph (PN), deutonymph (DN), tritonymph (TN) and adult (A) stages; all *n* = 4; different lowercase letters denote significant differences between different life stages (*P* < 0.05, ANOVA with *post-hoc* Tukey HSD Test); plots showed means ± (SEM).

In addition, the relative abundances of the main families and genera varied in patterns across the life cycle stages of *E. eharai* examined (Figures 2, 3; detailed lists of families and genera can be seen respectively in Supplementary Tables S5, S6). At the family level, the abundance of *Bacillaceae* was highest in the protonymph stage and lowest in the adult stage (*P* < 0.05, ANOVA with *post-hoc* Tukey HSD Test). The abundance of *Streptomycetaceae* was highest during the tritonymph stage and lowest in the adult stage, also with significant differences. The abundance of Alcaligenaceae remained consistent during the larval stage and significantly increased in the adult stage. The abundance of Tsukamurellaceae peaked significantly in the deutonymph stage and was lowest in the adult stage, with notable differences between these two stages. At the genus level, *Bacillus* was most abundant during the tritonymph stage and least abundant in the adult stage, with significant differences between these two stages. *Streptomyces* peaked in the protonymph stage and was lowest and significantly different in the adult stage. *Achromobacter* showed an opposite trend to *Streptomyces*, with significant differences between the protonymph and adult stages. *Tsukamurella* peaked in abundance during the deutonymph stage and was lowest in the adult stage (*P* < 0.05, ANOVA with *post-hoc* Tukey HSD Test).

### 3.4 Changes in structure of the bacterial communities throughout the life stages of *E. eharai*

**Figure 4:**
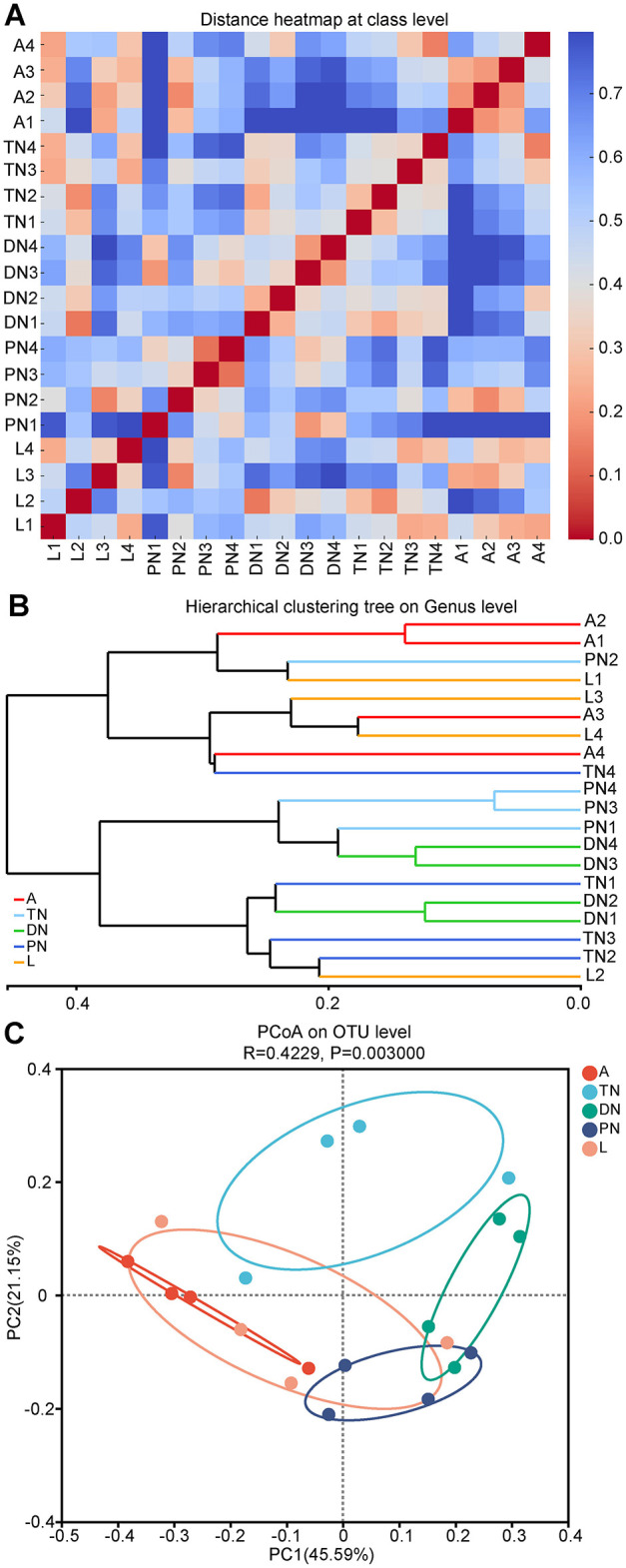
Bacterial community dissimilarity across five life stages of *E. eharai* based on bray curtis and weighted UniFrac metrics. **(A)** Sample distance heatmap analysis at class level. **(B)** Analysis of bray curtis method with arithmetic mean (UPGMA) based on hierarchical clustering at genus level. **(C)** Principal coordinated (PCoA) analysis at OTU level; the significance of variation in communities across four life stages was analyzed by analysis of similarities (ANOSIM) test with 999 permutations. L, PN, DN, TN and A indicated the larva, protonymph, deutonymph, tritonymph and adult stages, respectively.

Based on the composition of bacterial communities at various life stages, dissimilarities at the class, genus, and OTU taxonomic levels were further analyzed. A sample distance heatmap at the class level was generated using Bray-Curtis and weighted UniFrac metrics (Figure 4A). Additionally, a UPGMA analysis based on hierarchical clustering was performed at the genus level (Figure 4B), along with a PCoA analysis at the OTU level (Figure 4C). The heatmap results demonstrate notable differences in community structure among samples from various developmental stages. From an overall perspective, the community structures of the adult and larval stages were more similar to each other (except for sample L2), while those at the protonymph, deutonymph, and tritonymph stages showed lower similarity to those of the adult and larval stages. The genus-level hierarchical clustering analysis in Figure 4B further supported this finding, where samples from the adult and larval stages were closer in the dendrogram, while other life stages formed distinct branches. The PCoA analysis at the OTU level also revealed a similar trend, with samples from the adult and larval stages being closer to each other in the two-dimensional space, while other life stages were more distant (Figure 4C). The Analysis of Similarities (ANOSIM) results further confirmed significant dissimilarities in the bacterial communities between different life stages (*R* = 0.4229, *P* = 0.003; Figure 4C), especially between the adult and larval stages compared to other stages.

### 3.5 Microbial communities across the life stages of *E. eharai* based on PICRUSt prediction using the 16s rRNA gene marker

**Figure 5:**
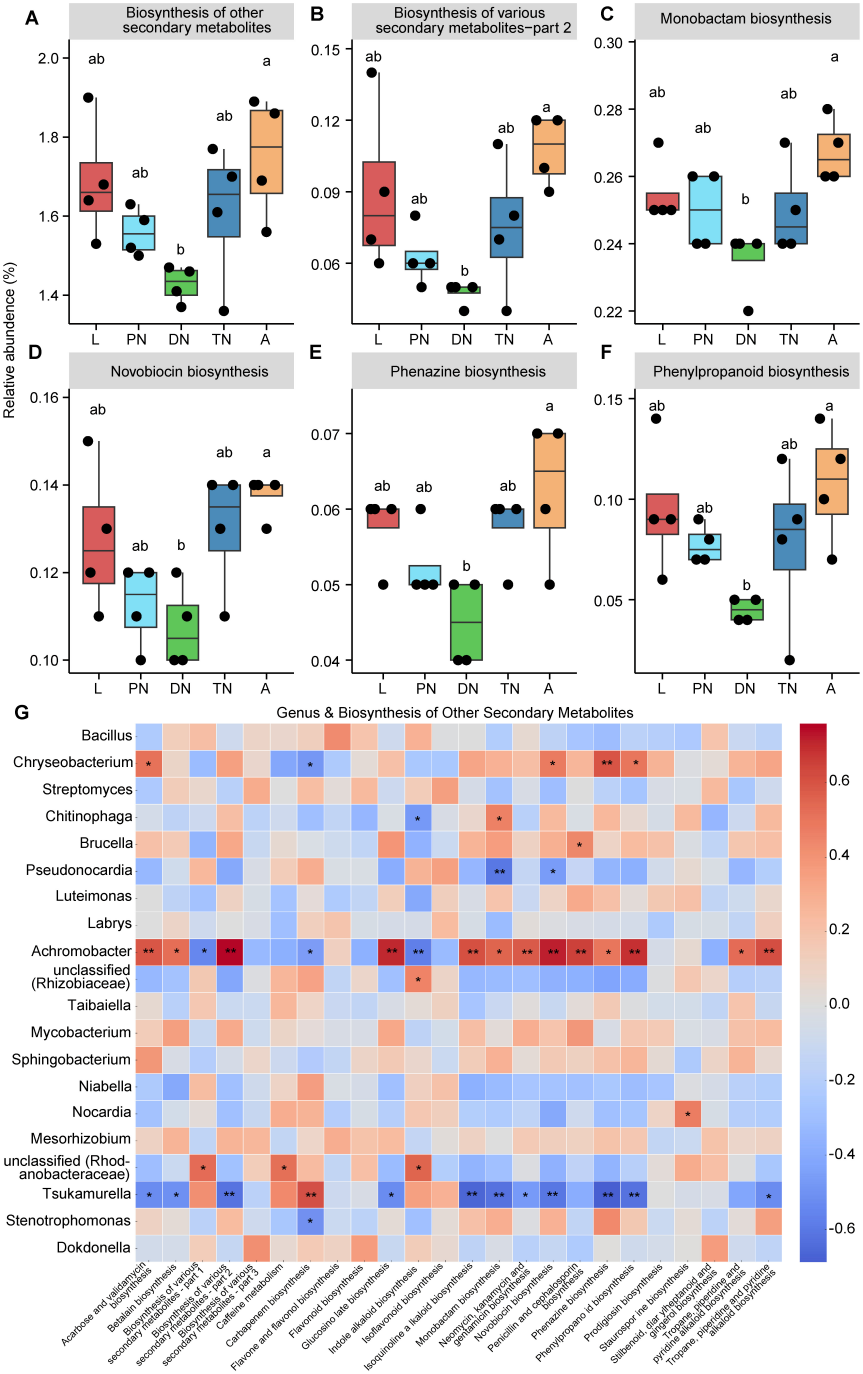
Dynamics of predicted KEGG pathways of “Biosynthesis of other secondary metabolites” and sublevel pathways across five life stages of *E. eharai*. **(A–F)** The “Biosynthesis of other secondary metabolites” pathway at level two **(A)** and five sublevel pathways at level three **(B–F)** significantly varied across the larva (L), protonymph (PN), deutonymph (DN), tritonymph (TN), and adult (A) stages; all *n* = 4; different lowercase letters denote significant differences between different life stages (*P* < 0.05, ANOVA with *post-hoc* Tukey HSD Test); plots showed means ± SEM. **(G)** Heatmaps based on Spearman's correlation analysis between the relative abundances of “Biosynthesis of other secondary metabolites” pathways and major genera; the red and blue represent positive and negative correlations between the relative abundances of pathways and bacterial genera, respectively; *Indicated *P* < 0.05, Spearman's correlation; **Indicated *P* < 0.01, Spearman's correlation.

**Figure 6:**
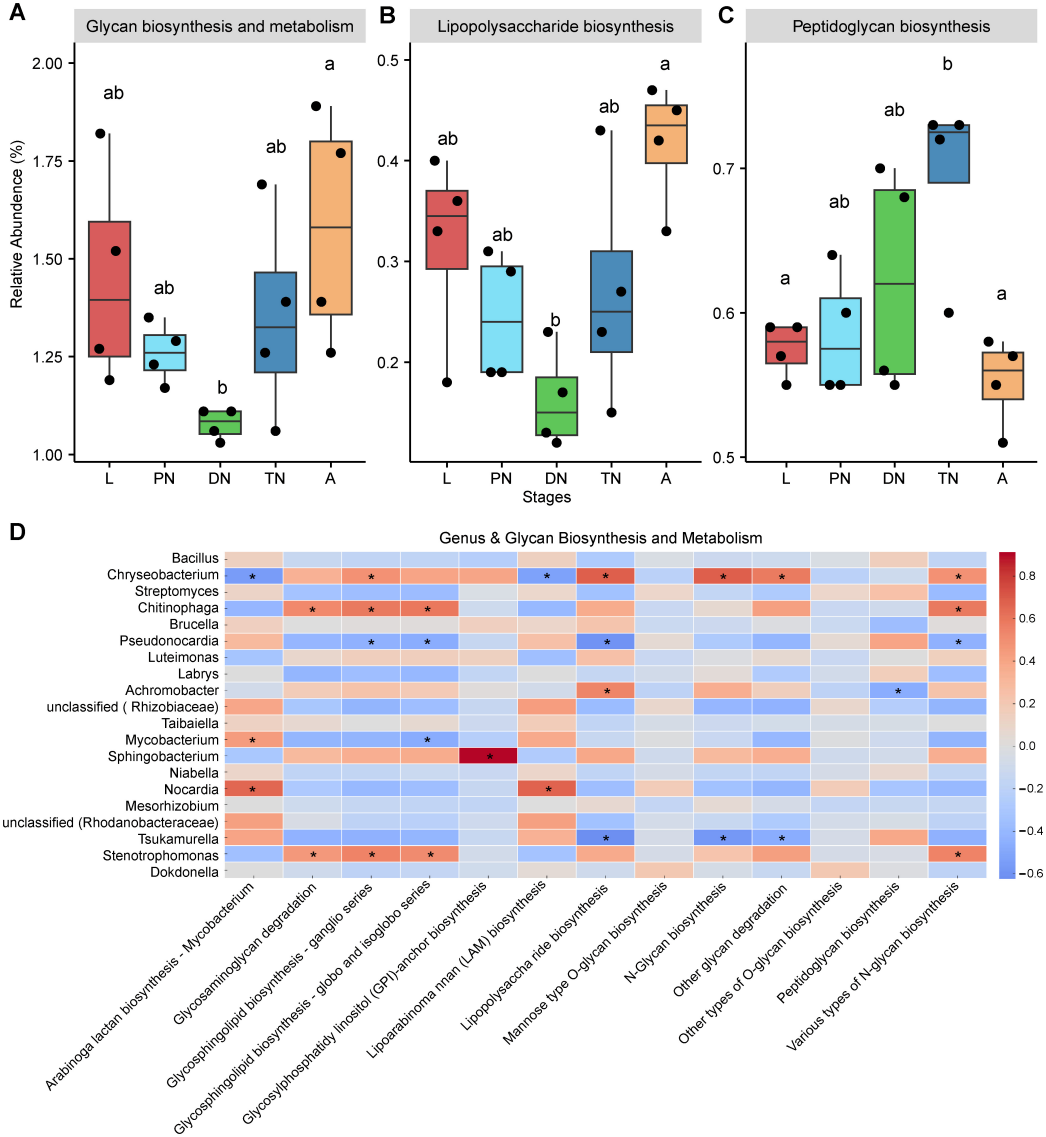
Dynamics of predicted KEGG pathways of “Glycan biosynthesis and metabolism” and sublevel pathways across five life stages of *E. eharai*. **(A–C)** The “Glycan biosynthesis and metabolism” pathway at level two **(A)** and two sublevel pathways at level three **(B,C)** significantly varied across the larva (L), protonymph(PN), deutonymph (DN), tritonymph (TN) and adult (A) stages; all *n* = 4; different lowercase letters denote significant differences between different life stages (*P* < 0.05, ANOVA with *post-hoc* Tukey HSD Test); plots showed means ± SEM. **(D)** Heatmaps based on Spearman's correlation analysis between the relative abundances of “Glycan biosynthesis and metabolism” pathways and major genera; the red and blue represent positive and negative correlations between the relative abundances of pathways and bacterialgenera, respectively; *indicated *P* < 0.05, Spearman's correlation.

Using PICRUSt to predict the functional profile of microbial communities based on the 16S rRNA gene marker, we identified 6, 46, and 389 KEGG pathways at levels 1, 2, and 3, respectively (see Supplementary Tables S7–S9 for detailed lists). Among the major KEGG pathways at level 2 (relative abundance > 1%), biosynthesis of other secondary metabolites (Figure 5A) and glycan biosynthesis and metabolism (Figure 6A) levels in the adult were similar to those in the larval, protonymph, and tritonymph stages, but significantly higher than in the deutonymph stage (*P* < 0.05). Meanwhile, the relative abundance trends of these pathways remained consistent in the larval and adult stages, and higher than in the other stages (Figures 5A, 6A). A similar pattern between larval and adult stages was also observed in five pathways including “Aging,” “neurodegenerative disease,” “drug resistance: antineoplastic,” “cardiovascular disease” and “cancer: specific types” (Supplementary Figure S3). In pathways of “nervous system” and “substance dependence,” the relative abundance in the deutonymph stage was significantly higher than in the adult stage, while the trends in the larval and adult stages remained consistent and were lower than those in the other life stages.

To study KEGG pathways related to host physiology, “biosynthesis of other secondary metabolites” and “glycan biosynthesis and metabolism” were selected for further analysis, as they have been identified to impact host physiological processes related to growth (Eilam et al., 2014; Funabashi et al., 2020; Martens et al., 2009). Within “biosynthesis of other secondary metabolites,” five pathways at level 3, including “biosynthesis of various secondary metabolite—part 2,” “monobactam biosynthesis,” “novobiocin biosynthesis,” “phenazine biosynthesis,” and “phenylpropanoid biosynthesis” showed a significant decrease in relative abundance in the deutonymph stage compared to the adult stage, with larval and adult stages always having higher relative abundances than other stages (Figures 5B–F; *P* < 0.05). In the area of “glycan biosynthesis and metabolism,” the adult stage showed a significant increase compared to the deutonymph stage in “lipopolysaccharide biosynthesis” pathway, while there was a significant decrease compared to the tritonymph stage in “peptidoglycan biosynthesis” pathway, with the larval and adult stages showing a similar trend in statistical significance (Figures 6B,C; *P* < 0.05). Detailed dynamics of the other six KEGG pathways are available in Supplementary Table S10.

To elucidate the relationship between changes in bacterial community composition and predicted functions, we performed a Spearman correlation analysis. The results showed that the relative abundances of *Chryseobacterium* and *Achromobacter* were positively correlated with most of the predicted “biosynthesis of other secondary metabolites” pathways, with 4 and 13 significant positive correlations, respectively. *Chryseobacterium, Chitinophaga* and *Stenotrophomonas* were positively correlated with half of the predicted “glycan biosynthesis and metabolism” pathways, with 5, 4 and 4 significant positive correlations, respectively (Figures 5G, 6D). In contrast, the relative abundances of *Pseudonocardia* and *Tsukamurella* were negatively correlated with most of the predicted “biosynthesis of other secondary metabolites” pathways, with 2 and 11 significant positive correlations, respectively. Moreover, these genera also showed negative correlations with most of the predicted “glycan biosynthesis and metabolism” pathways, with 4 and 3 significant negative correlations, respectively (Figures 5G, 6D).

## 4 Discussion

### 4.1 Bacterial community structure across developmental stages of oribatid mites and their functional roles

Our study is the first to investigate the patterns of bacterial community variation across different life stages of an oribatid mite species. We successfully reared *E. eharai* on a uniform diet of yeast in the laboratory, obtaining samples from all life stages, and then used high throughput 16S rDNA sequencing to compare the composition and structure of bacterial communities at different developmental stages of *E. eharai*. Bacteroidetes, Proteobacteria, Firmicutes, and Actinobacteria were identified as the four main phyla across different life stages of *E. eharai*, which is consistent with reported results on gut microbiota in soil-dwelling adult oribatid mites, where *Proteobacteria* and *Actinobacteria* were found to be the dominant phyla in the gut (Gong et al., 2018). Similar findings have also been reported for the gut microbiota of adult nematodes (*Caenorhabditis elegans*) (Berg et al., 2016). In addition, *Bacteroidetes, Proteobacteria*, and *Actinobacteria* were detected and found to be dominant in oribatid mites collected from the wild (Zhu et al., 2021). *Bacteroidetes* and *Proteobacteria* play a crucial role in promoting host development and the metabolism of key nutrients, enabling the host to obtain essential nutrients (Meng et al., 2019; Yao et al., 2019; Zhao et al., 2018). Therefore, these two phyla are probably significant in the growth and development of *E. eharai*. *Actinobacteria* appear to supplement nutrition and are required for normal growth. For example, early studies indicated that *Actinomycetes* spp. were present in oribatid mite species (Wolf and Rockett, 2009), and these bacteria can aid in chitin digestion and benefit the host by facilitating fungal digestion. Furthermore, *Streptomyces* spp. have been shown to produce chitin-degrading enzymes (Blaak and Schrempf, 1995). Our study shows that *Streptomyces* species are present in high abundance during the immature stages, with a significant decrease in abundance during the adult stage. This is likely because *E. eharai* has an immature digestive system during its juvenile stages and requires the assistance of *Streptomyces* to help digest food. Firmicutes play an important structural and physiological role in the guts of many insects, participating in the development and detoxification of toxic compounds, as has been demonstrated in some insects such as *Drosophila*, and bees (Engel and Moran, 2013; Mereghetti et al., 2017; Shao et al., 2017; Shin et al., 2011). The abundance of *Bacillaceae* in the phylum *Firmicutes* dominates in *E. eharai* fed on yeast, with the highest diversity observed at the protonymph stage and the highest abundance at the tritonymph stage. This may be related to the need for disease prevention during the nymphal stages. *Bacilli* have been reported to be commonly found in soil animals that feed on detritus, such as springtails, termites, isopods, myriapods, and earthworms, and are closely associated with the digestion of polysaccharides and aromatic compounds (Bouchon et al., 2016; Konig, 2006; Zimmer and Topp, 1998). Bacilli have also been found in many types of domestic mites (Hubert et al., 2016), and evidence has suggested that they likely provide nutrition to the mites (Hubert et al., 2012). Additionally, studies have found that the presence of *Bacillus subtilis* in the gut of *Caenorhabditis elegans* increases the nematode's resistance to Gram-positive pathogens, indicating that certain high-abundance bacterial communities of *Bacillaceae* can enhance host immunity, particularly during immature growth stages (Garsin et al., 2001; Ikeda et al., 2007). Thus, *Firmicutes* are likely to be very beneficial in maintaining the health of *E. eharai* during nymph stages. Our findings indicate that *Bacteroidetes* and *Proteobacteria* have the highest relative abundance in adult oribatid mites, whereas *Firmicutes* are least abundant in adults. Different dominant bacterial genera in the adult stages consistently show significant differences in relative abundance compared to a certain immature stage, possibly because the adult oribatid mites possess a more complex or developed digestive system. Furthermore, the association of *Firmicutes* with the physiological needs and mature immune systems of adults may rely more on other microbial phyla to maintain health, leading to varying degrees of difference (Douglas, 2015; Engel and Moran, 2013).

Endosymbionts are widely present in arthropods, with *Wolbachia* and *Cardinium* being the most extensively studied examples. They significantly impact the ecology and evolution of terrestrial arthropods by manipulating host reproduction, such as parthenogenesis, sex ratio distortion, infertility, and cytoplasmic incompatibility. (Ebrahimi et al., 2019; Lindsey et al., 2016; Provencher et al., 2005; Weeks and Breeuwer, 2001; Weeks and Stouthamer, 2004). Although previous studies have detected the presence of *Wolbachia* and *Cardinium* in various oribatid mite species (Konecka, 2022), these bacteria were not found in the parthenogenetic *E. eharai* specimens examined in our study. While *Wolbachia* and *Cardinium* are known to regulate host reproduction, many oribatid mite species do not harbor them. For instance, in a study of 20 suspected parthenogenetic oribatid mite species, only one was found to contain *Wolbachia* (Konecka and Olszanowski, 2015). Another study revealed that out of 15 *Damaeus onustus* samples collected from the same forest, only one was infected with *Wolbachia*, highlighting the generally low infection rates in oribatid mite species that are infected with (Konecka et al., 2020). Our study indicated that the population we tested was not infected with *Wolbachia* which is consistent with previous findings.

### 4.2 Bacterial diversity patterns in *E. eharai a*cross life stages

The bacterial diversity of *E. eharai* laboratory specimens fed on yeast, fluctuates across different life stages, with significant differences observed between the protonymph and tritonymph stages. This fluctuating trend is inconsistent with the declining diversity observed in other arthropods as they progress through their life stages, such as the citrus red mite (*Panonychus citri*) (Zhang et al., 2020), psyllids (*Diaphorina citri*) (Meng et al., 2019), ladybirds (*Illeis Koebelei*) (Yun et al., 2014), planthoppers (*Nilaparvata lugens* Stal, BPH) (Wang et al., 2020), and fruit flies (*Bactrocera minax*) (Yao et al., 2019). It is worth noting that bacterial diversity of the larval and adult stages of *E. eharai* are similar by calculating diversity indices, and cluster analysis showed that samples from these stages tend to cluster together, being more similar in composition than other stages. This is also inconsistent with the conclusions drawn for the aforementioned arthropods. This phenomenon may be due to larvae inheriting the main bacterial groups from the mother. The maternal microbial community can be transmitted not only through direct physical contact but also indirectly by influencing the genetic and epigenetic states of the eggs, thereby shaping the offspring's microbiome (Bright and Bulgheresi, 2010; Duron et al., 2008; Engel and Moran, 2013; Hurst and Frost, 2015; Jia et al., 2017; Perlmutter and Bordenstein, 2020). It is also possible that the similarity is due to the larvae and adult mites being fed the same diet of yeast and reared in the same environment.

### 4.3 Key bacterial genera and their roles in metabolic pathways during *E. eharai* development

Consistent with the results on diversity and composition, PICRUSt prediction results also show consistency in the dynamics of predicted KEGG pathways including “biosynthesis of other secondary metabolites,” “glycan biosynthesis and metabolism,” “aging,” “neurodegenerative disease,” “drug resistance: antineoplastic,” “cardiovascular disease,” “nervous system,” “cancer: specific types,” and “substance dependence,” where larval and adult stages are always consistent, and their relative abundances are always higher or lower than other life stages. This may be partly because the larval and adult stages share similar rearing environment and dietary habits, and could also be due to some microbes being vertically transmitted from the mother to the offspring (Bright and Bulgheresi, 2010). “Biosynthesis of other secondary metabolites” and “glycan biosynthesis and metabolism” show consistent dynamic changes across different life stages, with significant differences between the deutonymph and adult stages, which may be because adults and deutonymphs have significantly different physiological needs. The deutonymph stage is a transitional phase of development, during which they need to accumulate sufficient energy and nutrients for further growth and molting. At this stage, they are likely more reliant on basic metabolism and cellular repair processes to support the rapid development and changes in their bodies. Adults typically require more energy and nutrients to support reproduction and maintain their adult state, and secondary metabolites and glycan substances play important roles in these processes (Douglas, 2015). However, these factors were not directly measured in this study. Future research could incorporate detailed dietary intake analysis and anatomical examinations to further explore their potential impact. Heatmaps based on correlation analysis between metabolic pathways and major genera show that *Achromobacter* is significantly positively correlated with “biosynthesis of other secondary metabolites” pathways and *Chryseobacterium* is significantly positively correlated with “glycan biosynthesis and metabolism” pathways, while *Tsukamurella* shows significant negative correlation, proving that these three genera are one of the key factors dominating the changes in these two pathways. The relative abundance of *Achromobacter* and *Chryseobacterium* reach their peak during the adult mite stage, which may be related to the need for adults to absorb more nutrients to sustain their life activities. *Achromobacter* is a versatile bacterium, known to participate in various biochemical processes, including the decomposition of complex organic materials that help host nutrient absorption and energy conversion (Johnston et al., 2015; Traglia et al., 2012). *Chryseobacterium* is a Gram-negative bacterium that has the ability to produce antimicrobial substances, thereby protecting the host from infections (Chhetri et al., 2022), which may explain why these two genera are positively correlated with glycan biosynthesis and metabolism pathways, as these pathways are directly involved in the synthesis and metabolism of extracellular sugars. *Tsukamurella* is a typical slow-growing bacterium, often associated with the decomposition of difficult organic materials in ecosystems. These bacteria play crucial roles in the decomposition of complex organic compounds in the environment, characterized by slow metabolic activity and low resource demands (Khan and Yadav, 2004; Yassin et al., 1997). Pathways such as “biosynthesis of other secondary metabolites” and “glycan biosynthesis and metabolism” are typically associated with microorganisms that exhibit rapid growth and high energy requirements. The higher abundance of *Tsukamurella* during the deutonymph stage and its lowest abundance during the adult stage may be due to differences in energy demands between these two stages. However, this study did not directly measure energy requirements, metabolic rates, or resource competition across developmental stages, so this hypothesis requires further validation.

### 4.4 Limitations of laboratory cultivation and its impact on microbial community studies in oribatid mites

Artificial cultivation under laboratory conditions may influence the physiological state and behavioral performance of oribatid mites, such as reproduction, growth rate, and survival rate. These changes could indirectly affect the structure and function of microbial communities, thus the research results might not accurately reflect the true state of oribatid mites in their natural ecosystems. Moreover, in the wild, oribatid mites may encounter and consume a variety of food resources, including various organic debris, fungi, bacteria, etc., which provide more complex and diverse nutrients. Since yeast was used as the sole food source in the laboratory, this may have significantly influenced the composition and variation patterns of bacteria within oribatid mites and failed to simulate the rich nutritional and microbial diversity found in natural environments. Therefore, the study results cannot fully represent the microbial community structure and function of wild species in natural environments. Additionally, in our study, the strict control of experimental conditions limited the microbial diversity introduced through horizontal transmission. Therefore, we hypothesize that the microbiota inherited by larvae is likely closer to the maternal core microbiota. However, certain microbes may still be transmitted through shared food materials. As the microbiota of the egg stage was not analyzed in our experiment (primarily due to experimental feasibility constraints), the conclusion regarding maternal vertical transmission of microbes requires further validation. However, raising oribatid mites on the same diet in the laboratory also provides a relatively reliable basis for comparing the changes in gut microbiota across different stages. Compared to other soil animals, such as nematode and collembola, little is known about the impact of microbial communities on the performance and physiological composition of oribatid mites at different developmental stages, mainly because it is nearly impossible to obtain mite samples from all life stages in the wild, and the identification of immature stages of oribatid mites is also very challenging. Additionally, the current research on the types of food consumed by oribatid mites is very limited, making it difficult to know what they actually eat, hence replicating a wild environment for oribatid mites' cultivation has become a challenge. This study not only successfully reared oribatid mites but also provided valuable data on the composition and variation patterns of microbiota across all developmental stages of *E. eharai* when fed a diet of yeast, laying a foundational basis for further research on the interactions between oribatid mites and microbial communities.

## 5 Conclusion

In conclusion, this study revealed changes in bacterial community composition across different life stages of *E. eharai* when fed on yeast. There are few studies of the oribatid microbiome due to the difficulties inherent in rearing and maintaining different life stages in the laboratory. Our work is a first step to developing rearing and experimental methods to investigate changes in gut microbiota across life stages of oribatid mites. Given that we observed changes in gut flora in different life's stages, despite being fed the same diet, raises questions as to the role played by gut architecture, and the length of time the host spent at a given stage, in shaping gut flora.

The findings of this study show the importance of understanding the complex interactions between hosts and their microbial symbionts, especially how they jointly affect the host's physiological functions and adaptability. This work provides a basis for further research on oribatid mites.

Future work should focus on more detailed analysis of how the gut microbiome affects specific physiological processes of the host, as well as how they influence the host's response to environmental stress and ecological changes. Studies of oribatid mite in laboratory experiments that more closely simulate natural environments will help us to better understand the interactions between oribatid hosts and their gut flora and how these impact their survival.

## Data Availability

The datasets presented in this study are available in the online repositories at https://www.ncbi.nlm.nih.gov, accession number PRJNA1126081.
