# Supplementary material for: Shift patterns of internal bacterial communities across five life stages of laboratory-maintained Eremobelba eharai (Acari: Oribatida: Eremobelbidae)
Source: Front Microbiol. 2025 Mar 12;16:1512653. doi: 10.3389/fmicb.2025.1512653 (PMC11938428; doi:10.3389/fmicb.2025.1512653)
Supplement: Supplementary file 2 [file Data_Sheet_1.docx]

**Supplementary Methods**

The sample number is set as follows: 24 individuals are used at the larva stage; 20 individuals are used at the protonymph stage; 16 individuals are used at the deutonymph stage; 12 individuals are used at the tritonymph stage; 12 individuals are used at the adult stage.

**Fig. S1**

**
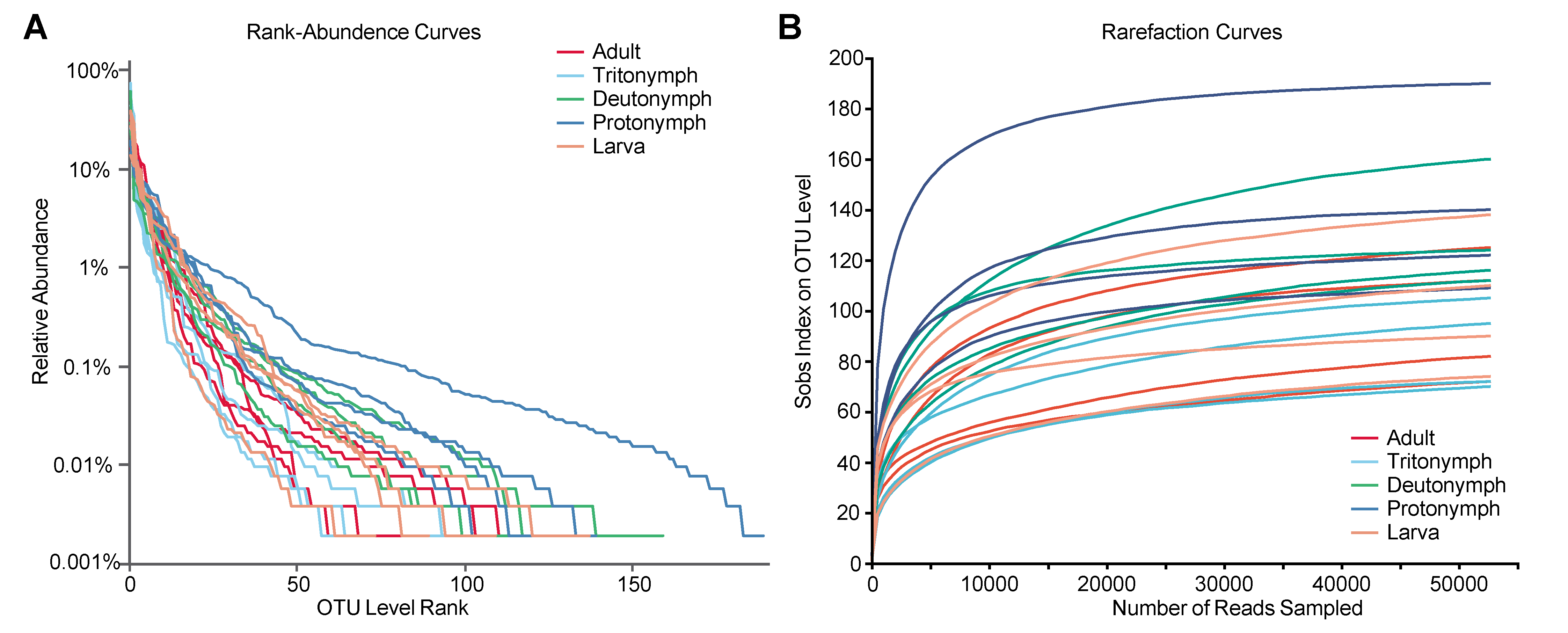
**

**Fig. S1. Rank-abundance curves (A) and rarefaction curves (B)** of bacteria based on bacterial OTUs at a similarity level of 97%.

**Fig. S2**

**
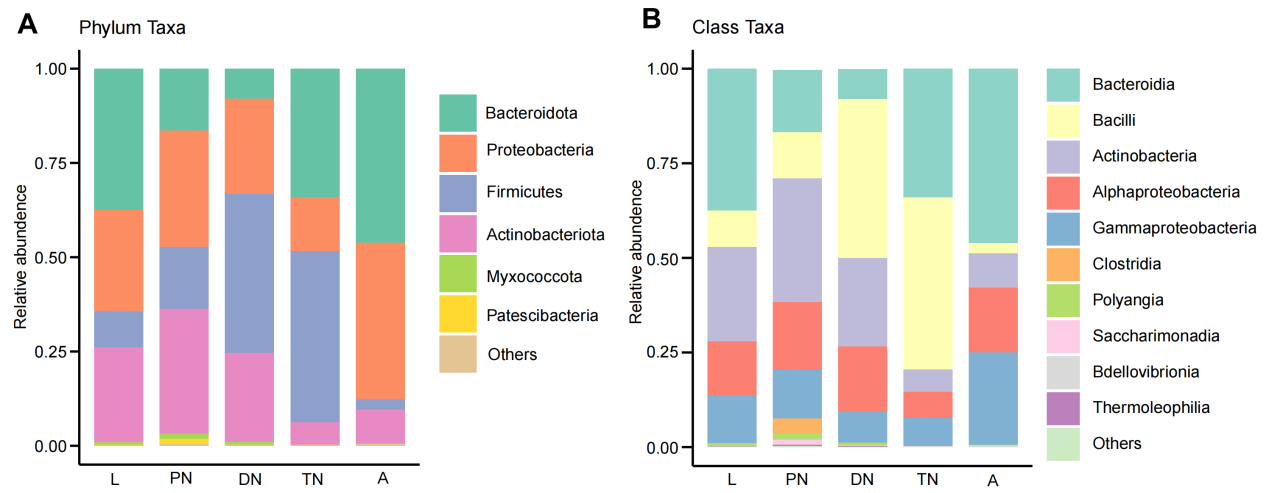
**

**Fig. S2. The Compositions of bacterial communities across five life stages of *E.eharai*.** Whole profiles of the relative abundances of the phylums (**A**) and classes (**B**) in each life stage; only taxa with a relative abundance > 0.1% in at least one sample were analyzed at phylum level, and only taxa ranked in the top 10 in relative abundance in at least one sample were analyzed at class level.

**Fig. S3**

**
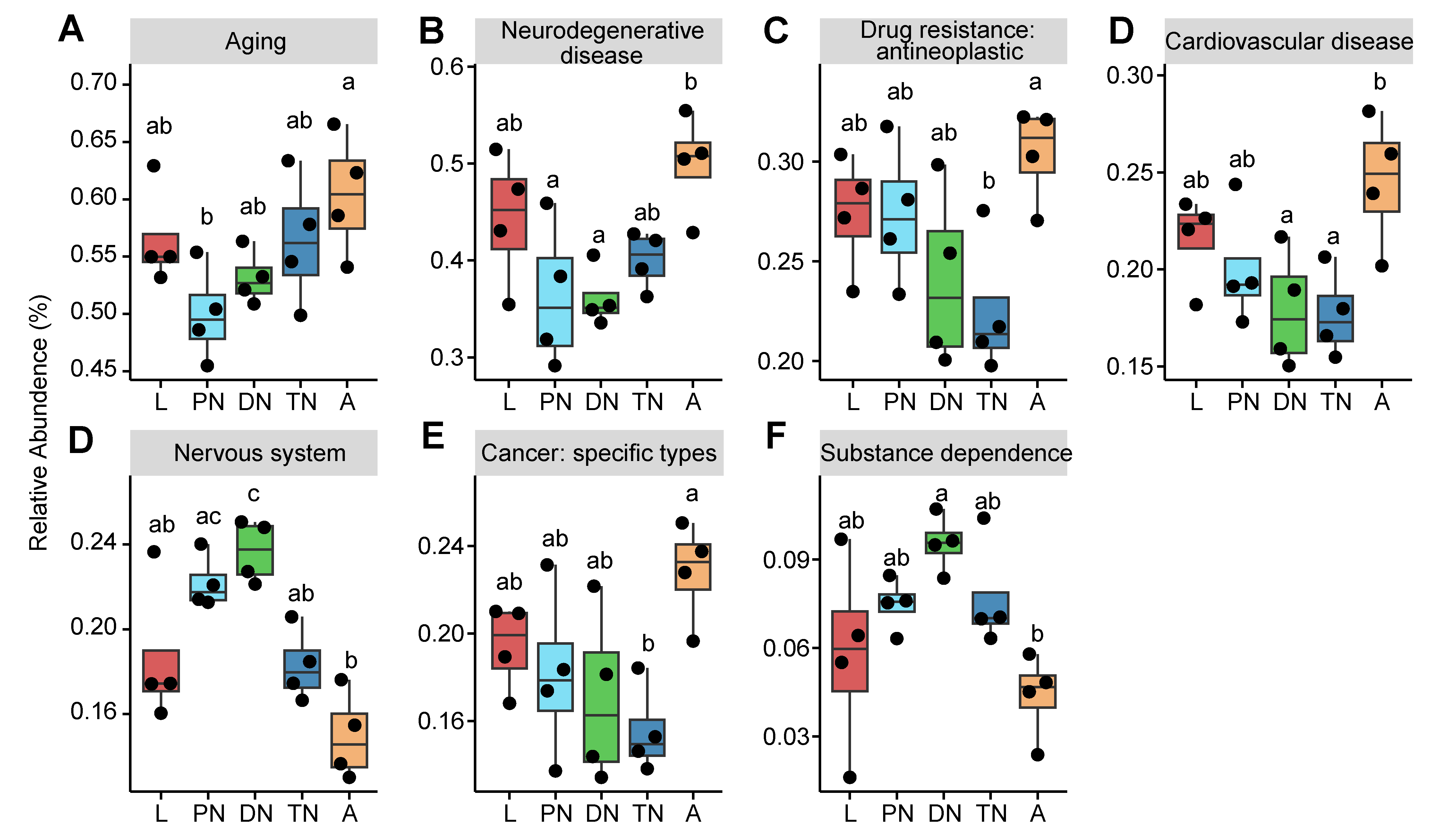
**

**Fig. S3. Dynamics of the predicted KEGG pathways at level 2 across five life stages of *E.eharai*.** All n = 4. Different lowercase letters denote significant differences between different life stages (*P < 0.05,* ANOVA with *post-hoc Tukey* HSD Test).
